# Features of tumor microenvironment in HER2-positive urothelial carcinoma and its implications for immunotherapy resistance

**DOI:** 10.1186/s12885-026-16157-1

**Published:** 2026-05-18

**Authors:** Jiazhi Mo, Jiayuan Chen, Mengnan Qu, Jinchang Wei, Yiran Huang, Li Zhou, Xieqiao Yan, Juan Li, Huayan Xu, Bixia Tang, Siming Li, Jun Guo, Xiaowen Wu, Xinan Sheng

**Affiliations:** Department of Genitourinary Oncology, Key Laboratory of Carcinogenesis and Translational Research (Ministry of Education/Beijing), Peking University Cancer Hospital & Institute, Beijing, China

**Keywords:** Urothelial carcinoma, HER2-positive, Tumor microenvironment, Immunofluorescence, Immunotherapy resistance

## Abstract

**Background:**

HER2 overexpression or ERBB2 amplification represents an important molecular feature of urothelial carcinoma (UC) and has been associated with differential responses to immune checkpoint inhibitors (ICIs). However, the tumor microenvironmental (TME) features associated with HER2-defined UC and their potential relationship with ICI responsiveness remain incompletely characterized.

**Methods:**

Tumor tissues from 52 UC patients treated with ICIs monotherapy at our center underwent multiplex immunofluorescence (IF) staining to evaluate immune cell infiltration and immune checkpoint protein expression. In parallel, transcriptomic and somatic mutation data from 408 UC cases in The Cancer Genome Atlas (TCGA) were analyzed. Differential gene expression and pathway enrichment analyses were performed based on HER2 copy number status (GISTIC score ≥ 2 for amplification). The xCell algorithm was used to estimate immune cell infiltration in bulk RNA-seq data.

**Results:**

In the institutional cohort, HER2-positive tumors showed numerically lower objective response rate (ORR) and disease control rate (DCR) than HER2-negative tumors, although these differences were not statistically significant. Multiplex IF analysis revealed reduced infiltration of CD4⁺ T cells, CD8⁺ T cells, and CD20⁺ B cells in HER2-positive tumors. PD-1 and STING expression were also lower in HER2-positive tumors, whereas LAG-3, TIM-3, CTLA-4, and PD-L1 showed non-significant downward trends. In the TCGA-BLCA cohort, ERBB2-amplified tumors showed downregulation of immune-related pathways and reduced inferred infiltration of multiple immune cell populations, including T cells, dendritic cells, B cells, NK cells, and macrophages.

**Conclusions:**

HER2-positive UC in the institutional IHC-defined cohort and ERBB2-amplified UC in the TCGA cohort were associated with reduced adaptive immune infiltration and attenuated immune-related signaling. These findings support the presence of an immune-cold phenotype in HER2-defined UC and provide hypothesis-generating evidence for further investigation of biomarker-guided combination strategies integrating HER2-targeted therapy with immune checkpoint blockade.

**Supplementary Information:**

The online version contains supplementary material available at 10.1186/s12885-026-16157-1.

## Introduction

Urothelial carcinoma (UC) is the tenth most common malignancy worldwide, with approximately 573,000 new cases and 213,000 deaths per year globally. Both its incidence and mortality are rising year by year [1]. UC can occur throughout the urinary tract, including the renal pelvis, ureter, bladder (the most common site), and urethra.

The *ERBB2*gene, which encodes human epidermal growth factor receptor 2 (HER2), is located on chromosome 17. Alterations of HER2 — most commonly protein overexpression or gene amplification — are among the most frequent molecular abnormalities in UC. The HER2 protein consists of an extracellular ligand-binding domain, a transmembrane domain, and an intracellular tyrosine kinase domain [2]. Overexpression or amplification of *ERBB2* can lead to constitutive activation of HER2 signaling through autophosphorylation of cytoplasmic tyrosine residues, triggering multiple downstream pathways such as RAS/MAPK and PI3K/AKT. This drives a cascade of signaling events that ultimately activate nuclear oncogenes (e.g., *c-Fos, c-Jun*), promoting tumor cell proliferation, survival, and progression [3–5].

Multiple studies have reported high rates of HER2 positivity in UC (IHC 2 + or 3 + in ~ 36–52% of cases) [6]. In a Chinese cohort of 284 UC patients, 44% were HER2-positive (IHC 2 +/3 +) [7]. This finding aligns with recent data: for example, a 2024 MSKCC study reported a 52% HER2 positivity rate among 202 advanced bladder cancer patients [8].

With the advent of immune checkpoint inhibitors (ICIs) and anti-HER2 antibody–drug conjugate (ADC) in recent years, the survival outlook for advanced UC has been improving. However, not all subgroups benefit equally. The POLARIS-03 trial reported a 26% overall objective response rate (ORR) with PD-1 inhibitor monotherapy in previously treated advanced UC, with a median overall survival (OS) of 14.4 months [9]. Notably, a subgroup analysis showed that none of the 9 patients with HER2 gene amplification responded to PD-1 blockade, suggesting that high HER2 expression may adversely affect immunotherapy efficacy. Indeed, prior studies have hinted that HER2 pathway activation could modulate the tumor microenvironment (TME) in ways that promote immune evasion [10]. These observations raise the question of how HER2-positive tumors interact with the immune system and whether their immune microenvironmental features may be associated with reduced responsiveness to ICIs.

At the same time, emerging clinical evidence suggests that dual targeting of HER2 and the immune axis can yield synergistic anti-tumor effects in UC. A recent phase III trial (RC48-C016) demonstrated that first-line treatment with the HER2-targeted ADC disitamab vedotin plus the PD-1 inhibitor toripalimab significantly prolonged Progression-free survival (PFS) and OS in patients with HER2-expressing advanced UC, compared to standard chemotherapy [11]. The combination therapy achieved an ORR of 76.1%, far higher than the 50.2% ORR with chemotherapy, with a more favorable safety profile. These results, now published in *NEJM*, firmly establish HER2 as a therapeutic target in UC and indicate that HER2 blockade can markedly enhance immunotherapy efficacy in this setting. Beyond prospective trials, real-world data also suggest that not all patients benefit equally from HER2-ADC plus PD-1 blockade, underscoring the need for biologically grounded biomarkers to guide combination strategies [12]. However, the underlying mechanisms remain to be elucidated – in particular, how HER2 overexpression/amplification shapes the TME to influence immune responses and therapeutic outcomes.

Based on these observations, we hypothesized that HER2-defined UC is associated with an immune-cold TME characterized by reduced adaptive immune infiltration and attenuated immune activation, which may contribute to the limited efficacy of ICI monotherapy in this subgroup. To test this hypothesis, we used two complementary approaches. First, we performed multiplex immunofluorescence (IF) analysis in an institutional cohort of 52 UC patients treated with ICI monotherapy to compare immune cell infiltration and checkpoint-associated marker expression between IHC-defined HER2-positive and HER2-negative tumors. Second, we analyzed the TCGA-BLCA cohort to evaluate transcriptomic pathways, somatic mutation features, and computationally inferred immune infiltration according to ERBB2 copy-number status. This integrated approach was designed to identify HER2-associated immune microenvironmental features and to generate hypotheses for future biomarker-guided combination strategies.

## Methods

### Experimental design

This study consisted of two complementary components. The first component was a retrospective institutional tissue-based analysis of UC patients treated with ICI monotherapy, in which HER2 status was assessed by IHC and immune microenvironmental features were evaluated by multiplex IF. The second component was a public multi-omics analysis of the TCGA-BLCA cohort, in which tumors were stratified according to ERBB2 copy-number status to examine transcriptomic pathways, somatic mutation features, and computationally inferred immune infiltration. The institutional cohort and TCGA cohort were analyzed separately because HER2 status was defined by different assays in the two datasets.

### Clinical response assessment

We retrospectively identified 52 patients with advanced UC at our center. No formal sample size calculation was performed because this was a retrospective exploratory study. The institutional cohort included all eligible advanced UC patients treated with ICI monotherapy at our center between October 2018 and October 2024 who had available pretreatment tumor tissue and evaluable HER2 IHC and multiplex IF data. Therefore, the sample size was determined by case availability rather than prospective statistical power calculation.

Radiographic tumor responses were evaluated from medical records according to RECIST v1.1 criteria. ORR was defined as the proportion of patients achieving a complete response (CR) or partial response (PR). Disease control rate (DCR) was defined as the proportion achieving CR, PR, or stable disease (SD). PFS was measured from ICIs treatment initiation to radiographic disease progression or death.

HER2 status in tumor samples was determined by immunohistochemistry (IHC) on pretreatment tissue. IHC scoring followed standard criteria based on intensity and completeness of membranous staining in invasive tumor cells: 0 (no staining or < 10% of cells with any weak, incomplete staining), 1 + (≥ 10% with weak, incomplete staining), 2 + (≥ 10% with weak to moderate complete membrane staining, or < 10% with strong complete staining), and 3 + (≥ 10% with strong, complete membrane staining) [13].

In this study, HER2 terminology was used according to the assay and cohort analyzed. In the institutional cohort, HER2 status was defined by IHC. Tumors with IHC 2 + or 3 + were classified as HER2-positive, whereas tumors with IHC 0 or 1 + were classified as HER2-negative. In the TCGA-BLCA cohort, HER2/ERBB2 status was defined by copy-number alteration, with ERBB2 amplification defined as a GISTIC copy-number score ≥ 2. Accordingly, the terms “HER2-positive” and “HER2-negative” refer to the IHC-defined institutional cohort, whereas “ERBB2-amplified,” “HER2 CNV-high,” “non-amplified,” or “HER2 CNV-low” refer to TCGA-based copy-number analyses.

Patients were stratified by HER2 status for comparisons. ORR and DCR between groups were compared using Fisher’s exact test. Kaplan–Meier curves for PFS were plotted and compared by log-rank test. A two-sided *P* < 0.05 was considered statistically significant.

### Tissue immune profiling by multiplex IF

Formalin-fixed, paraffin-embedded tumor tissue blocks from the 52 patients were obtained, and 4-μm sections were cut for multiplex IF staining. Each case’s section underwent IHC to confirm HER2 status as described above, then multiplex IF to characterize immune infiltrates. We employed tyramide signal amplification (TSA)-based multiplex IF technology to simultaneously detect multiple immune markers on a single tissue Sect [14]. Primary antibodies and optimal dilutions were as follows: CD8 (cytotoxic T cells; 1:3000, ServiceBio, GB12068), CD4 (T helper cells; 1:1000, ServiceBio, GB1358), FOXP3 (regulatory T cells; 1:2000, ServiceBio, GB112325), CD20 (B cells; 1:200, ServiceBio, GB11540-50), CD68 (macrophages; 1:3000, ServiceBio, GB113150), CD163 (M2 macrophages; 1:3000, ServiceBio, GB11380), CD56 (natural killer cells; 1:600, ServiceBio, GB12041), PD-1 (1:2000, ServiceBio, GB15338), PD-L1 (1:1000, ServiceBio, GB14132), CTLA-4 (1:200, Santa Cruz, sc-376016), LAG-3 (1:200, Abcam, ab209236), TIM-3 (1:200, ZENBIO, 222,772), and STING (1:2000, ServiceBio, GB11197). Staining was performed sequentially for different markers using distinct fluorophores, with intermediate antibody stripping as needed, according to manufacturer protocols. Nuclei were counterstained with DAPI. Multicolor fluorescent images were scanned and acquired for analysis.

For image quantification, viable tumor regions were selected based on corresponding hematoxylin and eosin morphology and HER2 IHC-stained sections, while areas of necrosis, hemorrhage, poor staining quality, tissue folding, and extensive non-tumor tissue were excluded. Three to five representative non-overlapping high-power fields were analyzed for each case. Image analysis was performed using ImageJ by investigators blinded to clinical response data. Marker-positive cell density was calculated as the number of positive cells per unit area when individual positive cells could be reliably identified. For markers with diffuse membranous, cytoplasmic, or area-based staining patterns, positive area or mean fluorescence intensity was quantified. The current analysis focused on overall immune marker abundance within selected tumor regions and did not separately quantify intratumoral and stromal compartments; therefore, compartment-specific spatial conclusions were not made. For each marker, the density of positive cells (or positive area) within tumor regions was measured and recorded. Mean values were compared between HER2-positive and HER2-negative groups using Student’s *t*-test (for approximately normally distributed data) or the Mann–Whitney *U* test (for non-parametric data). Graphical visualizations were prepared with GraphPad Prism.

### TCGA data acquisition and processing

We obtained public multi-omics data for bladder urothelial carcinoma (BLCA) from TCGA to complement our tissue findings. RNA sequencing data, copy-number variation data, and clinical information for bladder UC were obtained from The Cancer Genome Atlas project TCGA-BLCA through the Genomic Data Commons portal (https://portal.gdc.cancer.gov). Somatic mutation data and tumor mutation burden (TMB) values for these cases were obtained from the cBioPortal database and the R package TCGAmutations(v0.4.0) [15]. We focused our analysis on *ERBB2*(HER2) gene copy number variation (CNV) status. Using GISTIC 2.0, we defined HER2 amplification as a GISTIC copy number score ≥ 2 [16]. Among the 408 TCGA cases with available data, 51 (12.5%) were classified as HER2 CNV-high (amplified), while the remainder were HER2 CNV-low (non-amplified). Clinical characteristics (age, sex, clinical stage, TNM stage) were compared between the HER2 CNV-high and CNV-low groups using chi-square or Fisher’s exact tests for categorical variables and t-test for continuous variables.

### Genomic analysis of mutations and TMB

Somatic mutation analysis was performed in the subset of TCGA-BLCA cases with available matched mutation annotation and ERBB2 copy-number classification. This subset included 14 ERBB2-amplified/HER2 CNV-high tumors and 113 non-amplified/HER2 CNV-low tumors. Because of the limited number of ERBB2-amplified cases with complete mutation data, mutation frequency, TMB, and somatic interaction analyses were considered exploratory and descriptive.

Using the TCGA somatic mutation data, we compared the mutational landscape of ERBB2-amplified vs non-amplified UC. TMB for each tumor was calculated as the total number of non-synonymous mutations per megabase. TMB distributions were compared between groups by Wilcoxon rank-sum test. We also identified the most frequently mutated genes in each subgroup and visualized them via oncoplots using the maftools R package (v2.18.0). Differences in mutation frequency for the top mutated genes were assessed by Fisher’s exact test, with *P* < 0.05 considered significant. Additionally, we performed a somatic interaction analysis to detect patterns of co-occurring or mutually exclusive mutations. The co-mutation analysis was conducted with maftools to highlight pairs of genes that were significantly co-mutated or exclusively mutated in either group.

### Differential gene expression and pathway enrichment

To identify transcriptomic differences linked to HER2 status, we performed differential gene expression analysis on the TCGA RNA-seq data. We compared gene expression between the HER2 CNV-high (amplified) group (n = 51) and the HER2 CNV-low group (n = 357). Using the DESeq2 method, we obtained a set of differentially expressed genes (DEGs) with a false discovery rate (FDR) adjusted *P*(padj) < 0.05 and absolute log2 fold-change > 1 [17]. We generated volcano plots to visualize the upregulated and downregulated genes in HER2-high tumors.

Gene set enrichment analysis (GSEA) was then applied to the ranked list of expression changes to identify enriched hallmark pathways associated with HER2 amplification. Pathways with FDR q < 0.25 were considered significantly enriched. We focused in particular on immune-related pathways. Additionally, we specifically examined the expression of key immune-related genes (such as cytokines, chemokines, antigen presentation molecules, etc.) between the two groups.

### TCGA-based immune infiltration analysis

To complement the IF tissue analysis, we evaluated immune cell infiltration in the TCGA cohort via computational estimation. We applied the xCell algorithm, which infers the relative enrichment of various immune and stromal cell types from bulk gene expression data [18]. xCell scores for immune cell populations (including CD8 + T cells, CD4 + T cells, B cells, NK cells, dendritic cells, macrophages, etc.) were calculated for each tumor in the HER2 CNV-high and HER2 CNV-low groups. We then compared these scores between groups using t-tests and visualized differences with boxplots and heatmaps (using the ComplexHeatmap R package). In addition, Pearson correlation analysis was performed to assess the relationship between HER2 copy number level and the infiltration scores of T cell subpopulations (naïve, central memory, effector memory) derived from published gene signatures [19]. This allowed us to explore how HER2 amplification might correlate with specific T cell phenotypes.

### Statistical analysis

Continuous variables were summarized as medians with ranges or means with standard deviations, as appropriate. Categorical variables were summarized as counts and percentages. Comparisons between two groups were performed using Student’s t-test for approximately normally distributed continuous variables and the Mann–Whitney U test for non-normally distributed variables. Categorical variables were compared using the chi-square test or Fisher’s exact test, as appropriate. ORR and DCR were compared using Fisher’s exact test. PFS was estimated using the Kaplan–Meier method and compared using the log-rank test. Differential gene expression analysis was performed using DESeq2, with differentially expressed genes defined by adjusted P value < 0.05 and absolute log2 fold-change > 1. GSEA was performed using ranked gene lists, and pathways with FDR q value < 0.25 were considered enriched. For exploratory mutation analyses, mutation frequencies were compared using Fisher’s exact test, and findings were interpreted descriptively because of the limited number of ERBB2-amplified cases. All statistical tests were two-sided, and P < 0.05 was considered statistically significant unless otherwise specified.

## Results

### Impact of HER2 status on treatment response in UC patients receiving ICIs monotherapy

This study included 52 UC patients, all of whom had previously received ICIs monotherapy as part of their treatment regimen. The median age was 61 years (range: 35–78), with 73.08% male. Most patients (67.31%) had upper tract tumors. Based on IHC, HER2 expression levels were classified as 3 + (15.38%), 2 + (38.46%), 1 + (26.93%), and 0 (19.23%). HER2-positive (3 +/2 +) cases accounted for 53.85% and HER2-negative (1 +/0) cases accounted for 46.15%. Clinical and pathological characteristics are summarized in Table 1.

**Table 1:** Clinicopathological characteristics of patients included in the immunofluorescence analysis

Clinicopathological features	N (%)
Age at diagnosis	Median (range): 61 (35–78)
Sex	
Male	38 (73.08%)
Female	14 (26.92%)
Primary site	
Upper tract urothelial carcinoma	35 (67.31%)
Bladder cancer	17 (32.69%)
T stage	
T1	7 (13.46%)
T2	10 (19.23%)
T3	21 (40.39%)
T4	7 (13.46%)
Unknown	7 (13.46%)
N stage	
N0	15 (28.85%)
N1	8 (15.38%)
N2	23 (44.23%)
N3	3 (5.77%)
Unknown	3 (5.77%)
M stage	
M0	0 (0.00%)
M1	52 (100.00%)
HER2 expression by IHC	
0	10 (19.23%)
1 +	14 (26.93%)
2 +	20 (38.46%)
3 +	8 (15.38%)
HER2 status	
Positive (3 +/2 +)	28 (53.85%)
Negative (1 +/0)	24 (46.15%)

*Abbreviations*: *HER2* human epidermal growth factor receptor 2, *IHC* immunohistochemistry

**Fig. 1:**
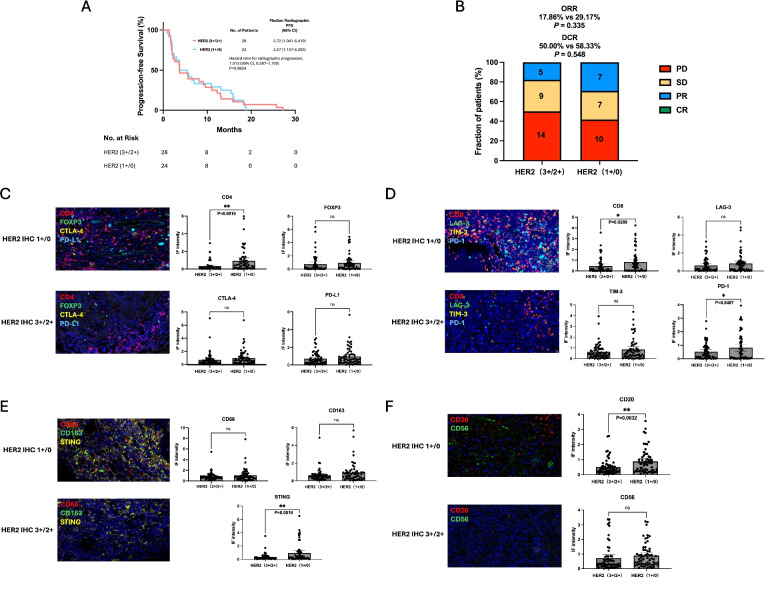
Impact of HER2 status on treatment response in UC patients receiving ICIs monotherapy and immune landscape of HER2-positive vs. HER2-negative UC analyzed by multiplex IF. HER2-positive tumors were defined as IHC 2 + or 3 +, whereas HER2-negative tumors were defined as IHC 0 or 1 + in the institutional cohort. **A** Best response to ICIs monotherapy by HER2 status. HER2-positive tumors had lower ORR (17.86% vs. 29.17%) and DCR (50.00% vs. 58.33%) compared to HER2-negative tumors, though the differences were not statistically significant. Response categories included progressive disease (PD), SD, PR, and CR. **B** Kaplan–Meier analysis of radiographic PFS. Median PFS was 3.72 months for HER2-positive and 4.57 months for HER2-negative patients (HR = 1.013, 95% CI: 0.587–1.700; *P* = 0.9624). **C** CD4⁺ T-cell infiltration was significantly lower in HER2-positive tumors (*P* = 0.0010), while no significant differences were observed in FOXP3, CTLA-4, and PD-L1 expression. **D** HER2-positive tumors exhibited reduced CD8⁺ T-cell infiltration (*P* = 0.0289) and PD-1 expression (*P* = 0.0487); LAG-3 and TIM-3 expression levels were similar between groups. **E** STING expression was significantly decreased in HER2-positive tumors (*P* = 0.0018); CD68⁺ and CD163⁺ macrophage infiltration showed no significant differences. **F** CD20⁺ B-cell infiltration was significantly lower in HER2-positive tumors (*P* = 0.0032), with comparable levels of CD56⁺ natural killer (NK) cells. IF intensity was quantified for each marker. *P* < 0.05 was considered statistically significant; *P < 0.05, **P < 0.01, ns: not significant

Among the 52 patients, HER2-positive tumors showed numerically lower ORR and DCR compared with HER2-negative tumors. The ORR was 17.86% in the HER2-positive group and 29.17% in the HER2-negative group, while the DCR was 50.00% and 58.33%, respectively. However, neither difference reached statistical significance (Fig. 1A). Median PFS was also numerically shorter in the HER2-positive group than in the HER2-negative group, but the difference was not statistically significant (Fig. 1B). These findings suggest a potential trend toward reduced benefit from ICI monotherapy in HER2-positive UC, but they should be interpreted cautiously because of the limited sample size.

### Tissue-based immune infiltration analysis using IF staining

IF analysis of tumor samples from 52 patients revealed that HER2-positive tumors had significantly lower infiltration of CD4 + T cells (*P* = 0.0010), CD8 + T cells (*P* = 0.0289), and CD20 + B cells (*P* = 0.0032) compared with HER2-negative tumors, suggesting reduced adaptive immune infiltration in HER2-positive tumors (Fig. 1C, D, and F). Other immune cells, including CD68 + macrophages, CD163 + M2 macrophages, CD56 + NK cells, and FOXP3 + regulatory T cells, also showed decreased fluorescence intensity in HER2-positive tumors, though without statistical significance. Moreover, immune checkpoint expression was lower in HER2-positive tumors, with PD-1 (*P* = 0.0487) and STING (*P* = 0.0018) significantly reduced (Fig. 1D, and E). LAG-3, TIM-3, CTLA-4, and PD-L1 also showed downward trends, but differences were not significant.

### Relationships between HER2 CNV level and somatic mutations

The TCGA dataset included 408 patients, comprising 51 in the HER2 CNV high group and 357 in the HER2 CNV low group. In the TCGA dataset, HER2 gene copy number amplification was not associated with gender (*P* = 0.1365), age (*P* = 0.3252), staging (*P* = 0.346), T stage (*P* = 0.5058), N stage (*P* = 0.1386), or M stage (*P* = 0.9374). The clinical characteristics of patients are presented in Table 1_S1.

**Fig. 2:**
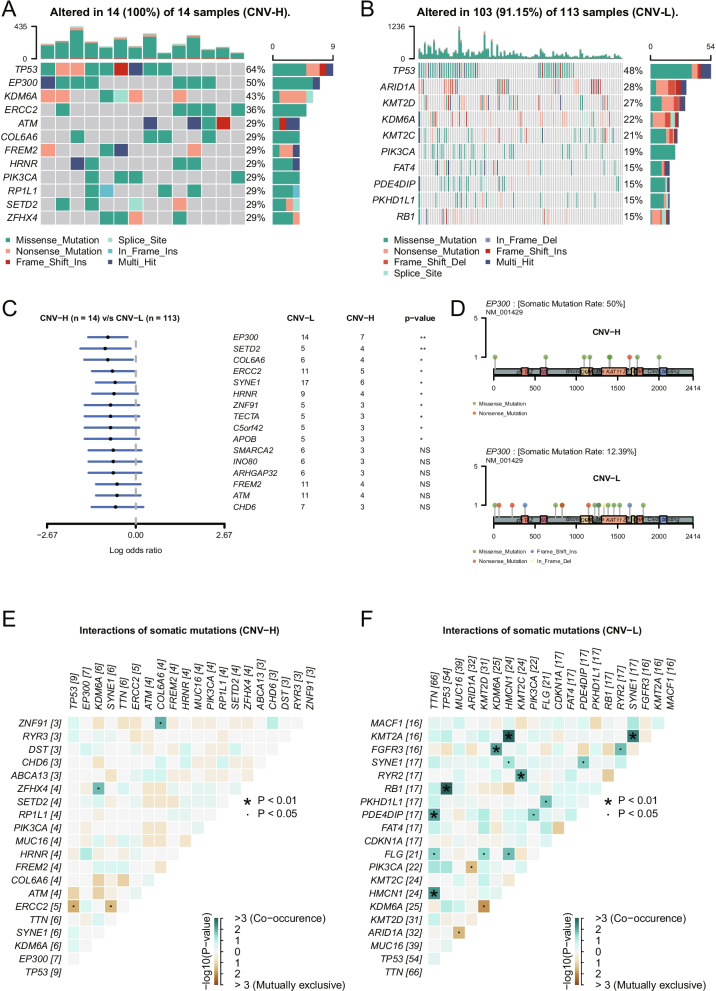
Relationships between HER2 CNV level and somatic mutations. Mutation analyses were performed in the subset of TCGA-BLCA cases with available matched somatic mutation and ERBB2 copy-number data. **A**-**B** Oncoplots of the mutated genes in HER2 CNV-high and -low groups. **C** Forest plot displaying differentially mutated genes between HER2 CNV-high and -low groups. **D** Protein mutation lollipop plots for EP300 between HER2 CNV-high and -low groups in BLCA. **E**–**F** Mutual exclusivity and co-occurrence of somatic mutations in HER2 CNV-high and -low groups. Because the ERBB2-amplified mutation subset was small, mutation frequency and somatic interaction results should be interpreted as exploratory

We next performed exploratory mutation profiling in TCGA-BLCA cases with available matched somatic mutation and ERBB2 copy-number data, including 14 ERBB2-amplified/HER2 CNV-high tumors and 113 non-amplified/HER2 CNV-low tumors. In the ERBB2-amplified subset, the most frequently mutated genes included *TP53*, *EP300*, *KDM6A*, and *ERCC2* (Fig. 2A), whereas *TP53*, *ARID1A*, *KMT2D*, and *KDM6A* were among the most frequently mutated genes in the non-amplified subset (Fig. 2B). ERBB2-amplified tumors showed a higher mutation count/TMB trend compared with non-amplified tumors (Fig. 2C-2D). Somatic interaction analysis suggested several potential patterns of co-occurring or mutually exclusive mutations (Fig. 2E-2F); however, given the small number of ERBB2-amplified tumors, these findings should be interpreted cautiously and require validation in larger genomic cohorts. Therefore, the mutation analysis was used primarily to provide exploratory genomic context rather than to infer HER2-specific mechanisms of immune evasion.

### Immune-associated transcriptomic features of ERBB2-amplified tumors

**Fig. 3:**
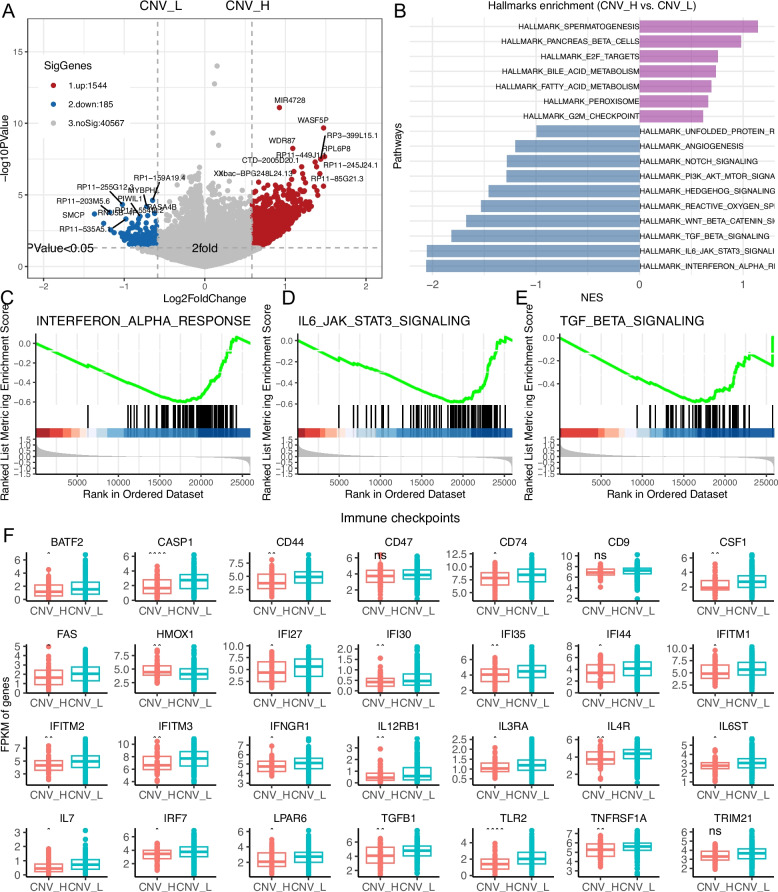
Transcriptome features associated with HER2 CNV level. HER2 CNV-high refers to ERBB2-amplified tumors defined by GISTIC copy-number score ≥ 2, whereas HER2 CNV-low refers to non-amplified tumors. **A** Differential expression genes (DEGs) in the TCGA-BLCA dataset between HER2 CNV-high and -low groups. **B** Differences in BLCA hallmark pathway activities between HER2 CNV-high and -low groups. The pink bars stand for the up-regulated pathways and the blue bars mean down-regulated pathways in HER2 CNV-high group. **C**-**E** Gene set enrichment analysis (GSEA) of hallmark gene sets that are significantly enriched in HER2 CNV-high vs CNV-low group. **F** Expression of immune checkpoints-related genes in BLCA. *, *P* < 0.05; **, *P* < 0.01; ***, *P* < 0.001; ****, *P* < 0.0001; ns, not statistically significant

HER2 CNV-high and -low groups' gene expression was compared using TCGA transcriptome data. To find the genes that were expressed differently in the HER2 CNV-high and -low groups, we conducted differential analysis. When compared to the HER2 CNV-low group, the volcano plot results revealed that the HER2 CNV-high group had 1,729 DEGs, of which 1,544 were upregulated and 185 were downregulated (Fig. 3A). The top 10 upregulated genes were *MIR4728*, *WASF5P*, *RP3 − 399L15.1*, *WDR87*, *RPL6P8*, *RP11-449J1.1*, *CTD-2005D20.1*, *RP11-245J24.1*, *XXbac-BPG248L24.13*, and *RP11-85G21.3*. The top 10 downregulated genes were *MYBPHL*, *RP1-159A19.4*, *RP11-255G12.3*, *PIWIL3*, *RP11-203M5.6*, *RP11-554I8.2*, *RNU5B-4P*, *SMCP*, *RP11-535A5.1*, and *RASA4B* (Fig. 3A). Using a GSEA, we investigated the underlying relationship between the HER2 CNV level and other biological processes. E2F targets and G2M checkpoint hallmark gene sets, as shown in Fig. 3B, were among the elevated hallmark gene sets in the HER2 CNV-high group that were eliminated. The set of E2F target genes, which are involved in many biological processes, was found to be significantly linked to the development of cancer and tumorigenesis [20–22]. GSEA showed that the HER2 CNV-high group had significantly more downregulated genes related to inflammatory-associated signaling or activities. In particular, a high level of HER2 CNV appeared to downregulate the interferon-alpha (IFN-α) response, IL6/JAK-STAT signaling pathway, and the transforming growth factor beta (TGF-β) signaling pathway (Fig. 3C-E).

Additionally, a number of immune-related genes were found to be downregulated, and the expression levels of these genes were compared between the HER2 CNV-high and -low groups. The findings showed that the HER2 CNV-high and -low groups differed significantly in the expression of the majority of immune-related genes (Fig. 3F). This result indicates a connection between these genes and the degree of HER2 CNV. Together, these findings suggest that ERBB2 amplification is associated with altered immune-related transcriptional programs in BLCA.

### Relationships between HER2 CNV level and immune infiltration

**Fig. 4:**
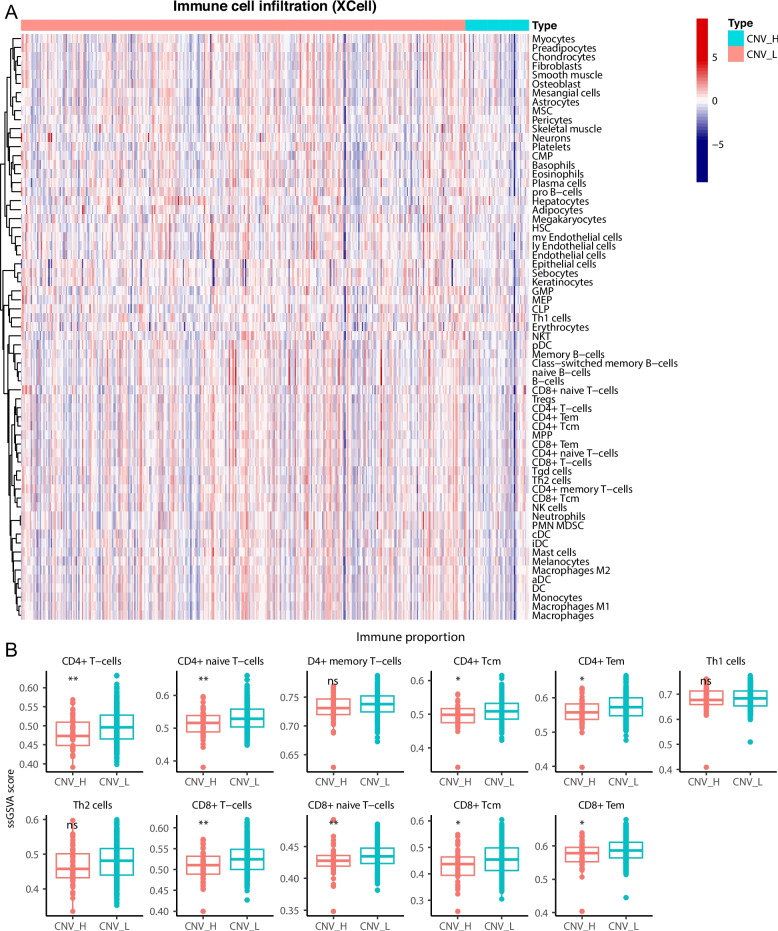
Analysis of immune cell infiltration between the HER2 CNV-high and HER2 CNV-low groups. Immune cell infiltration was computationally inferred from TCGA-BLCA bulk RNA-seq data using xCell and compared between ERBB2-amplified/HER2 CNV-high and non-amplified/HER2 CNV-low tumors. **A** Immune cell score heatmap by xCell algorithm. Red represents high expression/score, whereas blue represents low expression/score. **B** Comparison of various immune cell infiltration levels between HER2 CNV-high and -low groups. *, *P* < 0.05; **, *P* < 0.01; ***, *P* < 0.001; ****, *P* < 0.0001; ns, not statistically significant

We also used xCell analysis to examine the immune cell infiltration differences between the HER2 CNV-high and -low groups. Several cell types showed significantly lower infiltration in the HER2 CNV-high group, including CD4 + T cells, CD8 + T cells, dendritic cells (DC), B cells, NK cells, and macrophages (Fig. 4A and Fig. 4_S1). In the HER2 CNV-high group, immunoreactive cells (such CD8 + T cells and CD4 + T cells) were reduced, according to our research (Fig. 4A). These findings further supported reduced immune cell infiltration in ERBB2-amplified tumors. The phenotype of CD8 + T cells is essential for anti-tumor immunity, coordinating immunogenic cell death in malignancies via a number of methods. Recent research has shown that both central memory T (Tcm) and effector memory T (Tem) cells exhibit comparatively better antitumor immunity and durability [23]. Notably, we observed that patients in the HER2 CNV-high group exhibited lower infiltration of naïve CD8 + T cells, CD8 + Tcm cells, and CD8 + Tem cells. These findings suggest that ERBB2 amplification may be associated with reduced T-cell–related immune infiltration, which warrants further investigation in the context of ICI responsiveness (Fig. 4B). Additionally, there was a negative correlation between the HER2 CNV level and the infiltration levels of CD4 + memory T cells, CD4 + naïve T cells, CD4 + Tcm cells, and CD4 + Tem cells (Fig. 4B). Through a variety of molecular processes, CD4 + T cells aid in the initiation of a gene expression program in CD8 + T cells that improves CTL function and helps CTLs get past the barriers that normally impede antitumor immunity [24].

## Discussion

In this study, we integrated tissue-based multiplex IF profiling with TCGA-based transcriptomic and immune infiltration analyses to characterize immune microenvironmental features associated with HER2-defined UC. In the institutional IHC-defined cohort, HER2-positive tumors showed reduced infiltration of CD4⁺ T cells, CD8⁺ T cells, and CD20⁺ B cells, together with lower PD-1 and STING expression. In the TCGA cohort, ERBB2-amplified tumors showed downregulation of immune-related pathways and reduced inferred infiltration of multiple immune cell populations. These findings were directionally consistent across two independent analytical approaches and support the presence of an immune-cold phenotype associated with HER2-defined UC.

HER2 is a key molecular feature of UC and is widely used as a clinically meaningful biomarker, most commonly assessed by IHC. In UC, HER2 positivity is typically defined as IHC 3 + or IHC 2 + [25]. HER2 overexpression may arise from ERBB2 gene amplification or somatic alterations in regulators of HER2 expression (eg, c-Cbl), and transcriptional upregulation has been proposed as a major mechanism leading to increased HER2 mRNA and protein synthesis [26].

The prognostic significance of HER2 positivity in UC remains controversial. In our analysis, ERBB2 amplification was not associated with age, sex, or TNM staging parameters, and survival differences between ERBB2-amplified and non-amplified cases were not statistically significant.

The clinical outcome analysis should be interpreted with caution. Although HER2-positive tumors showed numerically lower ORR and DCR and a slightly shorter median PFS, these differences were not statistically significant. The lack of statistical significance may be partly explained by the small retrospective cohort size. Therefore, our clinical findings should be considered hypothesis-generating and require validation in larger prospective cohorts.

Nevertheless, prior studies have reported more aggressive clinicopathological behavior in HER2-positive tumors, including higher rates of vascular invasion and lymph node metastasis, and HER2 positivity has also been linked to metastatic patterns in other malignancies [27–30].

Given the oncogenic role of hyperactivated HER2 downstream signaling, therapeutic inhibition of HER2 is clinically important. Mechanistically, HER2 degradation can be regulated by c-Cbl-mediated ubiquitination and lysosomal trafficking, and additional transcriptional or cell-cycle regulators have been implicated in restraining HER2-driven tumorigenesis [31–34].

Notably, HER2 status determined by IHC (protein expression) and FISH (gene amplification) is not fully concordant and may have different predictive implications for treatment response. In a cohort of 42 UC patients evaluated by both assays, HER2 amplification was present in 31% of cases and was more frequent in IHC 3 + than in IHC 2 + tumors [35]. In the corresponding phase II study of trastuzumab deruxtecan, response rates varied across IHC/FISH-defined subgroups, and patients with HER2 amplification showed a trend toward greater benefit, suggesting that FISH-confirmed ERBB2 amplification may improve selection of UC patients likely to respond to HER2-ADC therapy [35].

Although platinum-based chemotherapy remains a cornerstone for advanced UC, combining HER2-targeted therapy with immunotherapy may further improve outcomes in HER2-positive disease. To better understand the biological basis for such combinations, we characterized the tumor microenvironment (TME) of HER2-driven UC. We observed enrichment of pathways including neuroactive ligand–receptor interaction, cytokine–cytokine receptor interaction, and PI3K-AKT signaling in ERBB2-amplified tumors, supporting a role for HER2-associated signaling programs in UC progression. In addition, stromal components such as endothelial cells, adipocytes, and fibroblasts represent key non-immune elements of the TME [36].

Both multiplex IF and TCGA analyses consistently demonstrated that high HER2 expression is associated with reduced infiltration of CD4 + T cells, CD8 + T cells, B cells, and M2 macrophages, together with a decreasing trend in multiple immune checkpoint-related molecules, including CTLA-4, PD-1, PD-L1, and STING. This pattern is consistent with an immune-cold phenotype in HER2-defined tumors and may provide a biological context for the limited activity of ICI monotherapy observed in some patients. However, because our study is observational and lacks functional validation, these findings should be interpreted as associative rather than causal.

Several immune-related features observed in this study may be relevant to reduced ICI responsiveness in HER2-defined UC, including reduced adaptive immune infiltration and lower STING expression. Prior studies have suggested that impaired dendritic cell-mediated antigen presentation, attenuated T-cell priming, and suppression of innate immune activation can influence responses to immunotherapy [37, 38]. In addition, HER2-related signaling pathways, including PI3K-AKT, have been implicated in immune modulation and tumor progression [39]. However, these potential links remain speculative in the present study and require functional validation.

Importantly, immune checkpoint expression should be interpreted cautiously, as bulk expression levels are influenced by both the number of infiltrating immune cells and checkpoint expression per cell. For example, PD-1 can be expressed across multiple activated immune cell types, while CTLA-4 and TIGIT show lineage-enriched expression patterns [40–44].

Consistent with prior observations that elevated HER2 may correlate with reduced immunotherapy responsiveness [9], resistance to PD-1 blockade may also involve low antigen immunogenicity, defective MHC-mediated antigen presentation, CD8 + T-cell exhaustion, and the establishment of an immunosuppressive TME [45–47]. Among extrinsic drivers, myeloid-mediated suppression is increasingly recognized as a dominant mechanism of immune escape. Tumor-associated macrophages (TAMs), particularly MARCO + TAMs, can impair neoantigen cross-presentation and restrain CD8 + T-cell cytotoxicity, thereby limiting ICIs efficacy [48]. In parallel, tumor-derived chemokines such as CCL2 and CCL3 may recruit immunosuppressive myeloid populations and further attenuate effector T-cell activity [49, 50].

Our findings are partly consistent with previous observations that HER2-altered tumors may exhibit reduced sensitivity to ICI monotherapy and altered immune microenvironmental features. The tissue-based multiplex IF results confirmed reduced adaptive immune infiltration in IHC-defined HER2-positive UC, while TCGA-based transcriptomic and immune deconvolution analyses extended these observations to ERBB2-amplified tumors. Therefore, the novelty of this study lies not in proposing HER2 as an immune-related biomarker for the first time, but in integrating clinical response, multiplex tissue immune profiling, and public multi-omics analyses to characterize immune-cold features associated with HER2-defined UC.

The clinical implication of these findings is that HER2 status may help identify a subgroup of UC with relatively reduced adaptive immune infiltration and attenuated immune activation. Such tumors may be less likely to respond robustly to ICI monotherapy, although this possibility requires prospective validation. The observation of an immune-cold phenotype also provides a biological rationale for combination approaches that incorporate HER2-targeted agents. HER2-directed ADCs may enhance tumor antigen release and modulate the immune microenvironment, thereby potentially improving the efficacy of PD-1/PD-L1 blockade. Taken together, we hypothesize that interrupting HER2-driven oncogenic signaling may help reprogram the TME toward a more immune-permissive state, providing a rationale for combining HER2-targeted agents with immune checkpoint blockade. Emerging clinical evidence supports this strategy, exemplified by the RC48-C016 trial showing that first-line disitamab vedotin plus toripalimab markedly improved PFS and OS compared with chemotherapy in HER2-expressing metastatic UC [11]. Our findings suggest that HER2-defined UC may be associated with a relatively immune-cold state, providing a biological context for future studies of HER2-targeted therapy combined with immune checkpoint blockade. Whether HER2-directed therapy can directly remodel the TME and enhance ICI responsiveness requires prospective and mechanistic validation.

Several limitations should be acknowledged. First, the institutional cohort was retrospective and included a relatively small number of patients, which limited statistical power for detecting differences in ORR, DCR, and PFS between HER2-positive and HER2-negative groups. Therefore, the clinical outcome findings should be considered hypothesis-generating rather than definitive. Second, HER2 status was defined by IHC in the institutional cohort but by ERBB2 copy-number status in TCGA analyses; although these approaches are biologically related, they are not fully interchangeable. Third, the multiplex IF analysis was based on selected tumor regions and did not separately quantify intratumoral and stromal compartments, limiting definitive spatial compartment-specific conclusions. Fourth, the TCGA immune infiltration analysis was computationally inferred from bulk RNA-seq data and may be influenced by tumor purity and transcriptomic deconvolution limitations. Fifth, the mutation analysis was limited by the small number of ERBB2-amplified tumors with complete matched mutation data. As a result, mutation frequency differences and somatic interaction patterns may be unstable and should be regarded as exploratory observations rather than definitive genomic features of HER2-amplified UC. Finally, no functional experiments were performed; therefore, the observed association between HER2/ERBB2 alterations and immune-cold features cannot establish direct causality. Further prospective studies and mechanistic experiments are needed to validate whether HER2-targeted therapy can remodel the TME and enhance sensitivity to ICIs.

In conclusion, HER2-positive UC in our institutional IHC-defined cohort and ERBB2-amplified UC in the TCGA cohort were consistently associated with reduced adaptive immune infiltration and attenuated immune-related signaling. These results support an immune-cold phenotype in HER2-defined UC and may help explain the limited activity of ICI monotherapy observed in some patients. However, our findings are associative and hypothesis-generating rather than causal. Larger prospective cohorts and mechanistic studies are needed to validate these observations and to determine whether HER2-targeted therapy can remodel the TME and enhance sensitivity to immune checkpoint blockade.

## Supplementary Information

Supplementary Material 1.

Supplementary Material 2.

## Data Availability

The datasets generated and/or analyzed during the current study are available from the corresponding author on reasonable request. Publicly available datasets (TCGA-BLCA) were used and can be accessed at https://portal.gdc.cancer.gov.
