# Supplementary figures and images for: Features of tumor microenvironment in HER2-positive urothelial carcinoma and its implications for immunotherapy resistance
Source: BMC Cancer. 2026 May 18;26:842. doi: 10.1186/s12885-026-16157-1 (PMC13366762; doi:10.1186/s12885-026-16157-1)

# Immune proportion

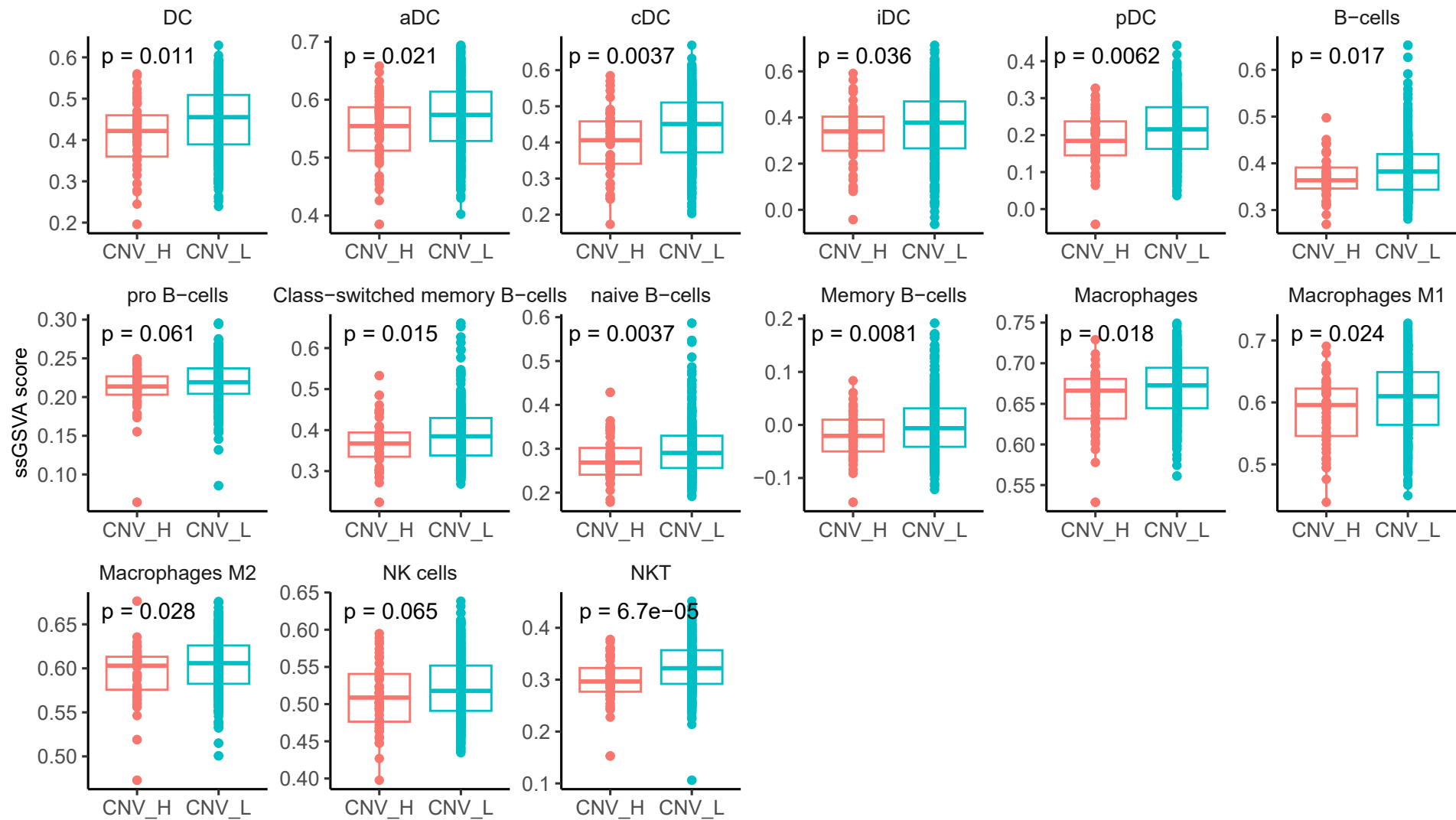

Supplement: Supplementary file 1 — Supplementary Material 1. [file 12885_2026_16157_MOESM1_ESM.pdf]
