# Supplementary material for: Features of tumor microenvironment in HER2-positive urothelial carcinoma and its implications for immunotherapy resistance
Source: BMC Cancer. 2026 May 18;26:842. doi: 10.1186/s12885-026-16157-1 (PMC13366762; doi:10.1186/s12885-026-16157-1)
Supplement: Supplementary file 2 — Supplementary Material 2. [file 12885_2026_16157_MOESM2_ESM.docx]

| **Characteristics** | **HER2 CNV high group (n = 51)** | | **HER2 CNV low group (n = 357)** | ***P* value** |
| --- | --- | --- | --- | --- |
| **Total patients** | 51 | 357 | |  |
| **Sex** |  |  | | 0.1365 |
| Female | 9 (17.65%) | 98 (27.45%) | |  |
| Male | 42 (82.35%) | 259 (72.55%) | |  |
| **Age** |  |  | | 0.3252 |
| < 45 years | 0 (0.00%) | 6 (1.68%) | |  |
| 45–65 years | 16 (31.37%) | 140 (39.22%) | |  |
| > 65 years | 35 (68.63%) | 211 (59.10%) | |  |
| **Clinical stage** |  |  | | 0.346 |
| I | 1 (1.96%) | 2 (0.56%) | |  |
| II | 13 (25.49%) | 117 (32.77%) | |  |
| III | 15 (29.41%) | 124 (34.74%) | |  |
| IV | 22 (43.14%) | 112 (31.37%) | |  |
| Unknown | 0 (0.00%) | 2 (0.56%) | |  |
| **T stage** |  |  | | 0.5058 |
| T1 | 1 (1.96%) | 3 (0.84%) | |  |
| T2 | 13 (25.49%) | 106 (29.69%) | |  |
| T3 | 21 (41.18%) | 172 (48.18%) | |  |
| T4 | 10 (19.61%) | 48 (13.45%) | |  |
| Unknown | 6 (11.76%) | 28 (7.84%) | |  |
| **N stage** |  |  | | 0.1386 |
| N0 | 23 (45.10%) | 213 (59.66%) | |  |
| N1 | 10 (19.61%) | 36 (10.08%) | |  |
| N2 | 9 (17.65%) | 66 (18.49%) | |  |
| N3 | 2 (3.92%) | 6 (1.68%) | |  |
| Unknown | 7 (13.73%) | 36 (10.08%) | |  |
| **M stage** |  |  | | 0.9374 |
| M0 | 25 (49.02%) | 171 (47.90%) | |  |
| M1 | 1 (1.96%) | 10 (2.80%) | |  |
| Unknown | 25 (49.02%) | 176 (49.30%) | |  |
| Abbreviations: HER2, human epidermal growth factor receptor 2; CNV, copy number variation. | | | | |
| **Table 1_S1: Characteristics of patients in the HER2 CNV high and low groups.** | | | | |
